# Understanding the Role of Fungi in Chronic Wounds

**DOI:** 10.1128/mBio.01898-16

**Published:** 2016-12-06

**Authors:** Matthew Malone, Hugh G. Dickson

**Affiliations:** a High Risk Foot Service, Liverpool Hospital, South Western Sydney Local Health District, Sydney, Australia; b LIVE DIAB CRU, Ingham Institute of Applied Medical Research, Sydney, Australia; c Molecular Microbiology Research Group, Microbiology and Infectious Disease, School of Medicine, Western Sydney University, Campbelltown Campus, Sydney, Australia; d Ambulatory Care Department, Liverpool Hospital, South Western Sydney Local Health District, Sydney, Australia

## LETTER

We thank Kalan and colleagues (1) for sharing their data on the role of fungi in chronic wounds, an area that has to date been understudied, with only one previously published article within the literature (2). Their study is therefore of great interest for clinicians in understanding the role of fungi in chronic wounds and in ascertaining whether alterations from antimicrobials to antifungals might improve outcomes.

The data presented are, however, difficult to interpret within the context of clinical management. First, Kalan and colleagues report the sampling of chronic wounds undertaken using the Levine technique with a swab. This culture method has been the subject of great debate in the diabetic foot arena, with opinions divided. Some expert groups promote tissue biopsy as the most appropriate sampling method for identifying pathogens of infection and for exploring both the microbiome and the role of biofilms (3, 6).

We note a previous study from the authors’ group suggesting good concordance between culture-independent swab samples (DNA sequencing) and tissue samples (4). However, the use of swab samples from superficial tissue makes it difficult to ascertain whether any fungi identified merely resided on wound surfaces as colonizers or whether the fungi were invasive and involved deeper tissue (this may suggest a more pathogenic involvement) (5).

Second, and more importantly, Kalan and colleagues report only on the ITS1 sequences (18S rRNA) and do not include bacterial or archaeal sequences (16S rRNA). In doing so, the clinical relevance of fungi in chronic wounds becomes lost. This is because, without identifying all the microorganisms within a wound (bacterial, fungal, archaeal), one cannot determine the overall microbial load for fungi or what their relative abundances are in relation to those of other microorganisms. This allows us to understand whether a microorganism is a dominant, major, or minor player. Therefore, no assumptions can be made on the community structure, and the “mycobiome” becomes clinically uninterpretable.
